# Identification and characterization of mouse otic sensory lineage genes

**DOI:** 10.3389/fncel.2015.00079

**Published:** 2015-03-19

**Authors:** Byron H. Hartman, Robert Durruthy-Durruthy, Roman D. Laske, Steven Losorelli, Stefan Heller

**Affiliations:** Department of Otolaryngology, Head and Neck Surgery, Stanford University School of MedicineStanford, CA, USA

**Keywords:** cochlea, vestibular, microarray, inner ear, transcriptome

## Abstract

Vertebrate embryogenesis gives rise to all cell types of an organism through the development of many unique lineages derived from the three primordial germ layers. The otic sensory lineage arises from the otic vesicle, a structure formed through invagination of placodal non-neural ectoderm. This developmental lineage possesses unique differentiation potential, giving rise to otic sensory cell populations including hair cells, supporting cells, and ganglion neurons of the auditory and vestibular organs. Here we present a systematic approach to identify transcriptional features that distinguish the otic sensory lineage (from early otic progenitors to otic sensory populations) from other major lineages of vertebrate development. We used a microarray approach to analyze otic sensory lineage populations including microdissected otic vesicles (embryonic day 10.5) as well as isolated neonatal cochlear hair cells and supporting cells at postnatal day 3. Non-otic tissue samples including periotic tissues and whole embryos with otic regions removed were used as reference populations to evaluate otic specificity. Otic populations shared transcriptome-wide correlations in expression profiles that distinguish members of this lineage from non-otic populations. We further analyzed the microarray data using comparative and dimension reduction methods to identify individual genes that are specifically expressed in the otic sensory lineage. This analysis identified and ranked top otic sensory lineage-specific transcripts including *Fbxo2, Col9a2*, and *Oc90*, and additional novel otic lineage markers. To validate these results we performed expression analysis on select genes using immunohistochemistry and *in situ* hybridization. *Fbxo2* showed the most striking pattern of specificity to the otic sensory lineage, including robust expression in the early otic vesicle and sustained expression in prosensory progenitors and auditory and vestibular hair cells and supporting cells.

## Introduction

**Figure 1:**
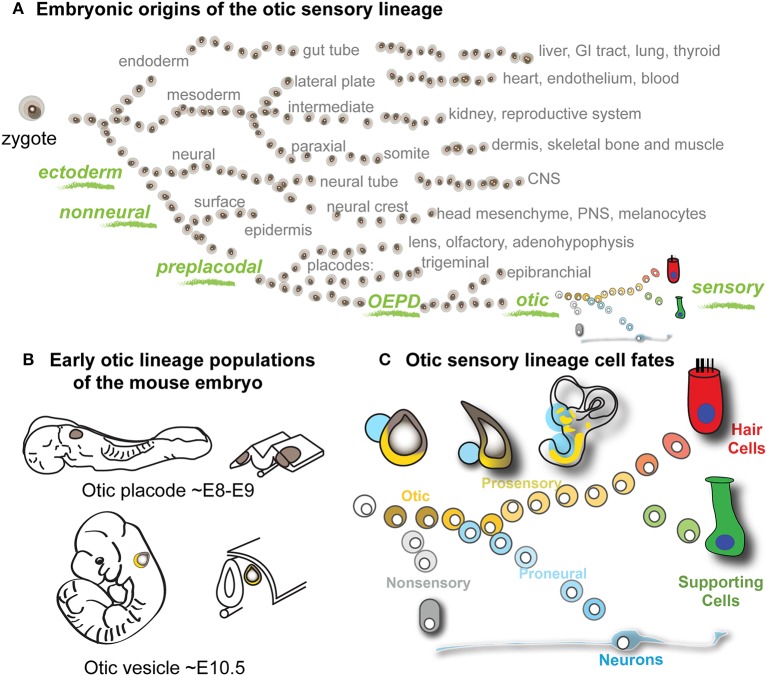
**Otic sensory development and project rationale**. **(A)** Schematic of the major developmental lineages of the vertebrate embryo. The otic sensory lineage is derived from the non-neural preplacodal domain of the ectodermal germ lineage (green). The otic-epibranchial domain (OEPD) gives rise to the otic placode, which invaginates to form the epithelium of the otic vesicle, from which the otic sensory populations differentiate. **(B)** The locations of early otic lineage populations of the mouse embryo are depicted at the placode and vesicle stages with corresponding embryonic ages indicated. **(C)** Model of otic lineage differentiation. Through progressive lineage bifurcations, cells of the otic vesicle give rise to hair cells, supporting cells and neurons, as well as non-sensory epithelial cells of the inner ear.

The sensory and neuronal cells of the vertebrate inner ear arise from the otic vesicle (OV), a spheroid epithelial structure formed during midgestation embryogenesis through invagination of the otic placode. Of all the developmental lineages of the vertebrate embryo, the otic sensory lineage has the unique capacity to give rise to auditory and vestibular hair cells, supporting cells, and neurons, all of which are essential for hearing and balance function (Figure 1A). The otic placode is derived from posterior preplacodal non-neural ectoderm, specifically within the otic-epibranchial domain (Chen and Streit, 2013). The process of otic induction from preplacodal ectoderm is initiated around embryonic day 8 (E8) in mouse. By E10.5 otic vesicle invagination has encapsulated the otic placode epithelia into a vesicle where subsequent processes of morphogenesis and differentiation give rise to cell fates of the inner ear, including neural, sensory (hair cells and supporting cells) and non-sensory fates (Figures 1B,C). A progressive lineage bifurcation model of otic differentiation is consistent with experimental observations and illustrates how pan-otic progenitor cells of the otic vesicle could give rise to non-sensory, neural, supporting cell, and hair cell populations (Figure 1C). Several signaling pathways including Fgf, Notch, and Wnt have been shown to be involved in specification of the otic placode as well as subsequent specifications of neuronal and prosensory populations (Liu et al., 2003; Jayasena et al., 2008; Dominguez-Frutos et al., 2009; Hartman et al., 2010; Hammond and Whitfield, 2011; Chen and Streit, 2013; Vendrell et al., 2013; Schlosser, 2014). Evolutionarily conserved transcription factors such as Eya1, Six1, Gata3, and Pax2/8 are expressed in the early otic sensory lineage and have been implicated in its specification and development (Xu et al., 1999; Zheng et al., 2003; Hans et al., 2004; Lillevali et al., 2006; Zou et al., 2006; Barrionuevo et al., 2008; Freter et al., 2012). Despite the unique nature of the otic sensory lineage, there are no known markers that unambiguously discriminate this lineage from others persistently through the course of development. The goal of this study was to apply a transcriptome-wide comparison to find such markers. Systematic transcriptome-wide identification of otic lineage distinguishing genes would contribute to our understanding of transcriptional states and gene regulation in inner ear development and function. Furthermore, classification of genes that enable rigorous identification of otic sensory lineage cells would greatly benefit *in vitro* studies of inner ear development and regeneration, which currently rely on combinatorial expression of transient non-specific markers (Oshima et al., 2010; Koehler et al., 2013).

Here we compare transcriptional states in three branches of the otic sensory lineage (early otic progenitors, sensory hair cells and supporting cells) to those of tissues broadly representing non-otic lineages of vertebrate development. We used multivariate analysis methods to identify correlations across ~25,000 probe sets that distinguish the otic sensory lineage from non-otic populations. Otic consensus genes were identified based on differential expression between otic (otic vesicle, hair cell, and supporting cell) and non-otic groups (whole embryos with otic regions removed and periotic tissues). Otic consensus scores and rankings for each probe were devised as a reduction of enrichment values in each of the three otic categories to further aid in identification of lineage specific genes. Our analyses ranked top otic lineage-specific transcripts and identified many novel genes expressed in early otic progenitors as well as sensory hair cells and supporting cells. We performed additional expression analyses on select genes using immunohistochemistry and *in situ* hybridization, which revealed patterns that concurred with the array data. *Fbxo2* showed the most striking pattern of specificity to the otic sensory lineage, including robust expression in the early otic vesicle and sustained expression in prosensory progenitors, and subsequently in auditory and vestibular hair cells and supporting cells.

## Materials and methods

### Mice

Embryos were collected from timed pregnant CD-1 dams (Charles River). Noon on the day of the vaginal plug was considered to be E0.5 and embryo ages were confirmed according to Theiler (Theiler, 1989). For postnatal mice, postnatal day 0 (P0) was defined as the day of birth. Mice were housed with the Stanford Department of Comparative Medicine and the Stanford University Administrative Panel on Laboratory Animal Care (APLAC) approved all procedures.

### RNA isolation from otic and non-otic tissue populations from E10.5 embryos

**Figure 2:**
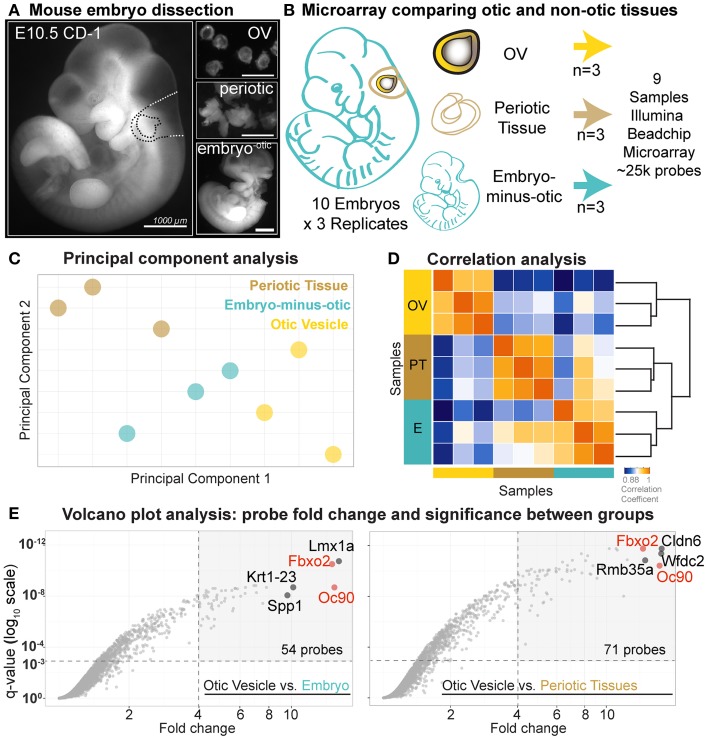
**Comparative early stage otic microarray**. **(A)** E10.5 mouse embryos were dissected into OV, periotic, and the whole embryo minus greater otic region. **(B)** Dissected tissues were collected as indicated and nine samples were processed for microarray. **(C)** Score plot of principal component analysis. Individual replicates for each of the three populations (OV, periotic tissue, embryo minus greater otic region) are projected onto the first two principal components. Color-code corresponds to tissue origin. **(D)** Correlation analysis between samples using Spearman's correlation coefficients as a measure of similarity. Replicates within one group of samples show higher correlation with each other than with samples of other tissue populations. **(E)** Volcano plots display corrected *p*-values (*q*-values) for each probe set as a function of associated fold change values between OV and each non-otic group. Fold change values calculated based on log2-normalized probe intensity values. Horizontal dashed lines indicate statistical significance level of *q* = 0.001. Vertical dashed lines indicate fold change = 4.

Three separate litters of E10.5 CD-1 embryos (Theiler Stage 16–17) were dissected in cold phosphate buffered saline (PBS) using fine forceps and separated into triplicate pools consisting of (1) 20 otic vesicles (OVs), (2) periotic tissue including mesenchyme and hindbrain, and (3) whole embryos minus the greater otic region (Figures 2A,B). RNA was isolated using the Nucleospin RNA XS Kit (Machery Nagel). RNA quality and concentrations were verified with Agilent BioAnalyzer RNA and Nanodrop spectrophotometer assays.

### Gene array hybridization

Each of the nine RNA samples was amplified using the Ambion Illumina RNA amplification kit with biotin UTP labeling. The Ambion Illumina RNA amplification kit uses T7 oligo(dT) primer to generate single stranded cDNA followed by a second strand synthesis to generate double-stranded cDNA, which is then column purified. *In vitro* transcription was done to synthesize biotin-labeled cRNA using T7 RNA polymerase. The cRNA was then column purified and a total of 750 ng was hybridized for each array using standard Illumina protocols with streptavidin-Cy3 used for detection. The MouseRef-8 v2.0 Illumina beadchip targets ~25,600 annotated RefSeq transcripts, over 19,000 unique genes, and enables the interrogation of eight samples in parallel. For practical purposes we will refer to the *transcriptome-wide* scope of data from this assay, although technically it is limited because some transcripts are not represented and some probe sets will perform better than others. Slides were scanned on an Illumina Beadstation and analyzed using GenomeStudio (Illumina, Inc.). The data discussed in this publication were deposited in NCBI's Gene Expression Omnibus, accession number GSE65843 (accessible at http://www.ncbi.nlm.nih.gov/geo/).

### Microarray data analysis

Differential microarray analysis was performed with the goal to identify genes enriched in early otic lineage cells as compared broadly to other developmental lineages. At E10.5 the mouse otic vesicle has fully invaginated from the head ectoderm and can be microdissected intact from the surrounding tissues (Figure 2A). Mouse embryos at E10.5 were dissected into three tissue groups, one otic and two non-otic. 20 otic vesicles were pooled for each OV sample and the remaining tissue of the embryos were dissected into two non-otic groups consisting of: (1) pooled periotic tissues including mesenchyme and dorsal neural tissue, and (2) whole embryos minus the greater otic regions (Figures 2A,B). We analyzed gene expression in triplicate samples for each group using MouseRef-8 v2.0 Expression Beadchip Microarrays (Illumina). To initially assess the quality of the data we performed principal component analysis (PCA) on all nine samples (Figure 2C). PCA reduces the high dimensionality of microarray data into a set of linearly uncorrelated variables called principal components, which are defined so that the first principal component retains the largest possible variance (Jollife, 2010). Projections on the first two principal components positioned samples of the same tissue origin in closer proximity with each other than samples from different origins (Figure 2C). As expected, transcriptome-wide expression profiles were more similar between replicates of each group. We also analyzed transcriptome-wide correlation between using Spearman's coefficients, which revealed intra-sample similarities and inter-sample differences (Figure 2D). Correlation coefficients and the structure of the associated dendrogram indicate that transcriptional profiles of otic vesicle cells are distinct from the two non-otic sample populations.

To compare gene expression in multiple otic sensory populations to non-otic populations, the data for the nine samples from E10.5 embryos described above were analyzed alongside equivalently generated array datasets for FACS-sorted neonatal cochlear hair cells and supporting cells generated in an earlier study (Sinkkonen et al., 2011). These included Illumina MouseRef-8 v2.0 datasets from P3 cochlear hair cells (HCs, *n* = 4, Atoh1-GFP+) and supporting cells (SCs, *n* = 2, Atoh1-GFP-/CD271L/CD146L/CD326+); NCBI Gene Expression Omnibus accession number GSE62582. GeneSpring 12.6 software and R 3.1.1 software packages (RStudio) were used for multivariate microarray data analysis. Data values were log2-transformed and quantile normalization was applied. Quantile normalization removes non-biological variance between arrays by making the distribution of probe intensities the same for each sample based on a normalization distribution chosen by averaging each quantile across all samples (Bolstad et al., 2003). Group means of expression and standard deviation (SD) were calculated for the five groups: E10.5 otic vesicle (OV), E10.5 periotic tissue, E10.5 embryo-minus-otic, and P3 hair cells (HCs) and supporting cells (SCs). Means of expression levels were compared between the otic groups and each of the two non-otic sample groups and fold change (fold Δ) values were calculated as the ratio of normalized mean intensities (for log2-transformed data, fold Δ = 2^∧^(A−B), where A and B represent log2-transformed normalized intensity values for two different samples). All probes were ranked in three categories based on fold Δ in expression value in the OV, HC, and SC groups vs. the maximum expressing non-otic group (periotic or embryo-minus-otic) for the given probe. Calculated *p*-values (moderated *t*-test, Smyth, 2004) were corrected for multiple comparisons using Benjamini and Hochberg's false discovery rate algorithm (termed *q*-values, Benjamini and Hochberg, 1995; Benjamini and Yekutieli, 2005). The R packages *FactoMinerR* and *gplots* were used to perform principal component analysis and visualize correlation and expression data in heat map format.

To identify otic vesicle specific genes we compared expression values between the E10.5 OV and the two non-otic populations individually (periotic, and embryo-minus-otic groups). Use of different non-otic reference populations affects the fold change values for some genes more than others. This is illustrated by the differences in probes with high fold change (fold Δ > 4) and high significance (*q* < 0.001) between comparisons (Figure 2E). For the OV vs. embryo-minus-otic comparison, 54 probes meet this cutoff, while 71 probes are found for the OV vs. periotic tissue. The probes with highest fold change in each category also change between comparisons, but some probes have high fold change in both, such as *Fbxo2* and *Oc90*, which are found in the top 5 probes for both comparisons. To rank genes based on the most conservative assessment of otic specificity, OV fold change values were determined using the highest non-otic intensity value for each probe (either the embryo-minus-otic, or the periotic tissue) (Supplemental File 1).

### Whole mount fluorescent immunohistochemistry

Whole embryos (E10.5–E12.5) or dissected neonatal cochlea tissues were fixed overnight at 4°C in either 4% paraformaldehyde (PFA) in phosphate-buffered saline at pH 7.4 (PBS), or *Glyofixx* (Thermo Fisher Scientific, Waltham, MA). Samples were washed in PBS then permeabilized with 2% TritonX100 in PBS for 1 h at room temperature. Samples were incubated in blocking solution (10% fetal bovine serum, 0.5% TritonX100, in PBS) overnight at 4°C. Primary antibodies were diluted in blocking solution and samples were incubated for three nights at 4°C with rocking. Samples were washed 4x in blocking solution for 1 h at room temperature, then overnight at 4°C, all with rocking. Species-specific Alexafluor 488-/568-/ or 633-conjugated secondary antibodies (Life Technologies) were diluted in blocking solution and samples were incubated for 2 nights at 4°C with rocking. Samples were washed twice at room temperature for 1 h in blocking solution, twice for 1 h in PBS, then overnight in PBS at 4°C, all with rocking. For optical clearing of whole embryos, samples were incubated at 4°C with rocking in Scale/A2 (Hama et al., 2011) for at least 5 days, with 2–3 changes of the Scale/A2 solution. For imaging, whole embryos were mounted in Scale/A2 between glass coverslips in imaging chambers consisting of 2–3 stacked 0.5 mm adhesive silicone spacers, modified from SecureSeal Hybridization Chambers (Grace Biolabs). Optically cleared embryos were imaged with a Zeiss LSM700 confocal microscope using a 10X or 20X objective to generate multiple z-stacks for coverage of the whole specimen. Maximum intensity z-projections were stitched together in Adobe Photoshop CS6 using the auto blend layers function.

### Cryosection fluorescent immunohistochemistry

Embryonic heads or neonatal inner ears were fixed overnight at 4°C in 4% PFA in PBS. Samples were washed in PBS, then cryoprotected through graded sucrose in PBS (10% sucrose, 20% sucrose, 30% sucrose), then embedded in OCT (Tissue Tek), frozen in a bath of ethanol and dry ice, sectioned at 12 μm, and mounted on Superfrost+ slides (Fisher Scientific). Slides with cryosections were then washed briefly in PBS and incubated with blocking solution (10% fetal bovine serum, 0.5% TritonX100, in PBS). Primary antibodies were diluted in blocking solution and incubated overnight at 4°C. Slides were then washed in PBS 3 × 10 min and incubated in species-specific Alexafluor 488-/568-/ or 633-conjugated secondary antibodies (Life Technologies). After immunostaining, slides were coverslipped in Fluoromount G (Southern Biotechnology, Birmingham, AL, USA) and imaged on a Zeiss LSM700 confocal microscope.

### Antibodies

The following primary antibodies were used for section and whole mount immunohistochemistry, at the dilutions indicated: rabbit anti-Fbx2 (1:300, Kato et al., 2005, gift of A. Kato and D. Bredt), goat anti-Sox2 (1:300, Santa Cruz Biotechnology, Sox2 Y-17, cat. no. SC-17320); goat anti-Sox10 (1:100, Santa Cruz Biotechnology, Sox10 N-20, SC-17342); mouse anti-Tuj1 (1:2000, Millipore, Ms X beta III tubulin, MAB5564); goat anti-Jag1 (1:300 Santa Cruz Biotechnology, Jag1 C-20, SC-6011); rabbit anti-COL9A2 (1:100, Atlas Antibodies, HPA056316), and rabbit anti-Pax2 (1:100, Covance).

### Paraffin section *in situ* hybridization

Digoxigenin-labeled probe was *in vitro* transcribed from a linearized complementary DNA clone corresponding to *Oc90* (MGC:59513 IMAGE:6334417). *In situ* hybridization was performed as previously described (Hartman et al., 2007). Briefly, whole embryos (E10.5 and E12.5) or half heads (E14.5 and older) were fixed overnight at 4°C in modified Carnoy's solution (60% ethanol, 11.1% formaldehyde (30% of 37% stock), 10% glacial acetic acid), dehydrated though an ethanol series, prepared for paraffin embedding, and sectioned at 10 μm. Slides were baked overnight at 68°C, dewaxed in xylene, rinsed in ethanol, and air-dried at room temperature. Overnight hybridization and subsequent washes were carried out at 68°C. Hybridized probe was detected using anti-digoxygenin alkaline phosphatase conjugated antibody (1:2000 dilution, Roche Biochemical, Indianapolis, IN, USA) and visualized with NBT/BCIP for a blue precipitate. After *in situ* hybridization, sections were postfixed in 4% PFA and coverslipped with Fluoromount G.

## Results

### Identification of genes distinguishing the otic vesicle from other lineages of the midgestation embryo

OV fold Δ values (see Methods) for the top 100 OV probes range from about 15- to 3-fold, and 47 probes had OV fold Δ values greater than 4 (Supplemental File 1). Low *q*-values indicated high statistical significance (*q* < 0.05) for the vast majority of the probes that ranked in the top 200 based on OV fold Δ (Table 1 and Supplemental File 1). Sensitivity and dynamic range are highly dependent on probe design and differ greatly between probes. This is reflected in the broad range of average OV intensity values among the top 30 OV probes (Table 1) and illustrates the necessity for differential comparison to reference populations. The MouseRef-8 Beadchip represents most genes with only a single probe (identified by Probe_ID, Supplemental File 1), however some genes are represented by more than one probe. The inclusion of two non-otic reference populations is useful for assessing dynamic range, estimating limit of detection and in some cases identification of the presence of non-otic expression sites. If an OV specific gene were not substantially expressed in other tissues then we would not expect to see differential expression between the two non-otic populations. Thus, periotic vs. embryo fold Δ values and statistical comparisons are included in Table 1 and Supplemental File 1.

**Table 1:** **Top 30 probes ranked by OV fold Δ vs. maximum non-otic population**.

**Gene**	**E10.5 otic vesicle**		**Otic vesicle vs. Max non-otic**			**Periotic vs. Embryo**	
	**Mean intensity**	***SD***	**OV Fold Δ**	***q*-value**	**OV rank**	**Fold Δ** (abs)	**UpReg (q > 0.05)**
***Oc90***	**10.97366167**	**0.419246795**	**15.316797**	**2.08E-09**	**1**	**1.132771783**	**NS**
***Fbxo2***	**10.87404333**	**0.072690153**	**14.622012**	**1.74E-12**	**2**	**1.023630354**	**NS**
*Lmx1a*	11.06934217	0.056113649	11.061509	3.38E-04	3	1.448138913	Periotic
*Krt1-23*	10.25196333	0.288855995	10.184871	2.04E-09	4	1.220269579	NS
*Spp1*	9.971883833	0.332888479	8.454517	1.67E-10	5	1.136606365	NS
*Cldn6*	13.18188567	0.089216859	7.5281053	3.69E-08	6	2.35525402	Embryo
Wfdc2^*^	13.53477267	0.213203	7.073592	5.33E-09	7	2.496286749	Embryo
*Gata3*	11.364921	0.142121662	7.0340185	1.49E-09	8	1.173139844	NS
***Col9a2***	**10.07369633**	**0.153509332**	**6.894713**	**1.49E-09**	**9**	**1.008878076**	**NS**
*Arhgef19*	9.768474333	0.09629171	6.341925	2.08E-09	10	1.218514062	NS
*Rbm35a*	12.29022383	0.143019237	6.3149257	6.10E-09	11	2.368308192	Embryo
*Plekhb1*	10.08439517	0.16829363	5.946511	6.30E-11	12	1.312607244	NS
*Hs3st1*	10.46399633	0.195136572	5.8575263	2.70E-09	13	1.048332013	No
*Rgcc*	10.77107167	0.161833702	5.628432	1.19E-08	14	1.990202734	Embryo
*Tbx2*	9.984906	0.297733516	5.626228	9.24E-08	15	1.702381408	Embryo
*Prss8*	10.105778	0.16620698	5.5848174	1.45E-07	16	1.290586405	NS
*Sh3gl2*	9.860674833	0.135098031	5.195888	8.67E-11	17	1.002118118	NS
Wfdc2^*^	9.499769333	0.302036446	5.1527414	2.46E-08	18	1.227582286	NS
*Marveld3*	9.041756667	0.133118972	5.006034	3.82E-09	19	1.094974091	NS
*Vwa2*	9.691604	0.165306445	4.864665	5.78E-08	20	1.376504348	NS
*Bdnf*	9.836624667	0.178453967	4.78742	8.09E-09	21	1.246577778	NS
*Espn*	10.76721233	0.250324449	4.7623343	1.19E-08	22	2.301431921	Embryo
*Plekha4*	9.914148	0.299348822	4.7167573	8.07E-09	23	1.282688658	NS
*Fgf10*	10.70834767	0.021669661	4.700219	2.49E-09	24	2.193222734	Embryo
*Car4*	10.77693067	0.369327027	4.63751	2.23E-06	25	1.171838835	NS
*Prr15*	9.310321667	0.225649081	4.6330085	2.20E-08	26	1.146866527	NS
*Col6a1*	10.12810567	0.204186116	4.622068	2.27E-09	27	1.199047241	NS
*Six1*	11.91456133	0.035842889	4.5915775	2.49E-09	28	1.492275252	Embryo
*Socs2*	12.39277283	0.234257353	4.547705	2.49E-09	29	1.097722074	NS
*Myo7a*	9.023172167	0.481244122	4.538013	1.31E-07	30	1.027957463	NS

OV rank was determined based on fold Δ in mean OV intensity vs. that of the maximum expressing non-otic group for a given probe. Genes that appear more than once (^*^) are represented by multiple probe sets and those in bold are included in validation studies in later sections. OV, otic vesicle; SD, standard deviation; NS, not significant.

The top-ranking OV probe was for the *Oc90* gene, with an OV fold Δ of 15.32 (*q* = 2.08E-09), while the probe for *Fbxo2* had an OV fold Δ value of 14.62 (*q* = 1.74E–12) (Figure 2E, Table 1, and Supplemental File 1). Both probes exhibited low non-otic group intensity values without significant differences between periotic and embryo-minus-otic samples (*Oc90* fold Δ = 1.13, *q* = 0.506; *Fbxo2* fold Δ = 1.02, *q* = 0.946, Supplemental File 1), suggesting that these two genes are otic-specific. Both of these genes were initially discovered based on high expression of RNA or protein in screens of rodent cochlea (Thalmann et al., 1997, 2003; Verpy et al., 1999). A probe for *Col9a2* (OV rank 9, OV fold Δ = 6.89, *q* = 1.49E-09) also had nearly identical intensities between the two non-otic populations (periotic vs. embryo fold Δ = 1.01, *q* = 0.981) suggestive of very low or undetectable expression in other lineages. Further discussion and analysis of expression patterns for *Oc90, Fbxo2*, and *Col9a2* are found below.

A difference in probe intensities between the two non-otic populations can be an indication of other regions of gene expression. For example, some probes had higher intensity in the periotic tissue group, which can indicate expression domains in the dorsal mesenchyme or neural tube. A probe for *Lmx1a* had a fold change in the OV vs. the rest of the embryo slightly higher than that of *Fbxo2*, however relatively high periotic tissue intensity put this probe in 3rd for the OV rankings (OV fold Δ = 11.06, *q* = 3.38E-04). The difference between the periotic group and embryo-minus-otic group intensities for the *Lmx1a* probe (fold Δ = 1.45, *q* = 0.005, Supplemental File 1) is consistent with the described expression of *Lmx1a* in OV as well as the dorsal midline (roof plate) of the developing neural tube (Failli et al., 2002; Nichols et al., 2008). Elevated probe intensity in the embryo-minus-otic group as compared to the periotic group was relatively common and is suggestive of gene expression in other ectodermal, endodermal, or mesodermal cell populations of the embryo. For example, *Cldn6*, *Wfdc2*, and *Rbm35a* (OV ranks: 6, 7, and 11, respectively), all have described expression patterns in the otic vesicle as well as endodermal tissues such as the branchial arches and pronephros (Kollmar et al., 2001; Anderson et al., 2008; Tamplin et al., 2008; Ohazama et al., 2010). Expression in these domains is reflected as significantly elevated intensities in the embryo-minus-otic group vs. the periotic group for all of these probes (Table 1 and Supplemental File 1). Two probes for *Fgf10* (OV ranks 24 and 37) were also elevated in the embryo-minus-otic group compared to the periotic group, reflective of *Fgf10* expression in the OV as well as limb bud mesoderm (Ohuchi et al., 1997; Mailleux et al., 2005). *Espn* (OV rank 22) was upregulated in the embryo compared to the periotic group, which is consistent with a well described pattern of Espin expression in early otic epithelia as well as brachial clefts, pharyngeal pouches and other embryonic epithelial tissues (Sekerkova et al., 2006).

### Comparison to neonatal hair cell and supporting cell gene array data and identification of otic consensus genes

**Figure 3:**
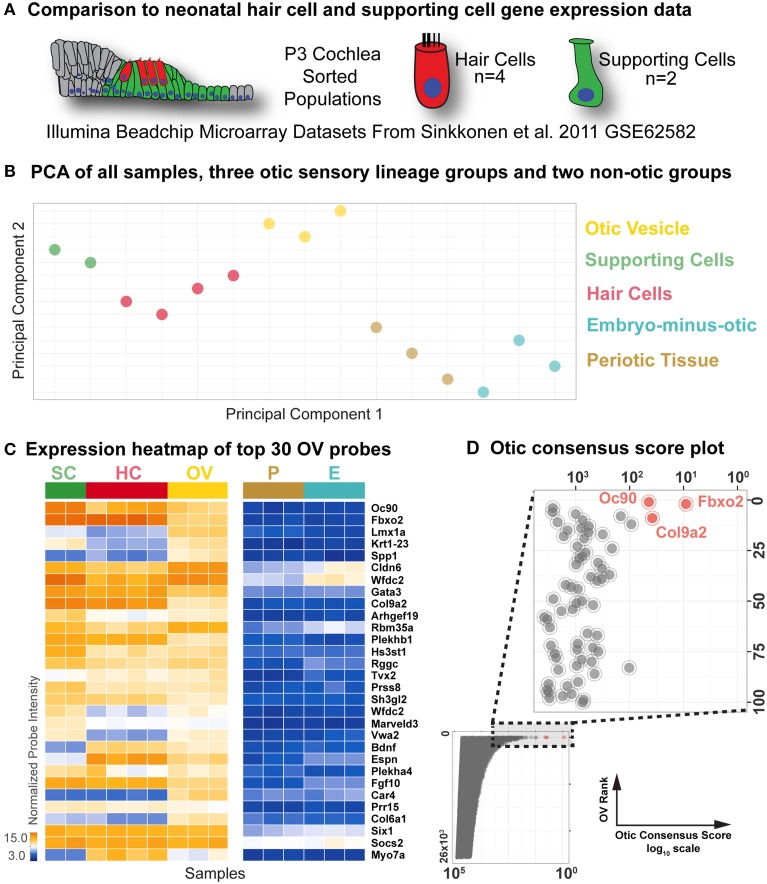
**Comparison to neonatal hair cell and supporting cell microarray data**. **(A)** Differential comparison was extended to an existing microarray dataset (GSE62582, Sinkkonen et al., 2011) consisting of flow-sorted hair cell and supporting cell populations from P3 cochlea. **(B)** Principal component analysis score plot of all samples, color-coded as indicated on the right. **(C)** Probe intensity heat map of top 30 OV ranked probes in all samples, indicated by group color in the top bar. Genes are ordered according to Table 1. **(D)** Otic consensus score plotted against OV rank for all probes, with an enlargement of the top 100 OV probes in the inset. The top three probes are indicated in red.

We next wanted to identify transcriptional features and individual genes that distinguish the otic sensory lineage from non-otic lineages throughout developmental time. To address this, we added two more otic populations to our comparative array analysis: hair cell (HC) and supporting cell (SC) RNA samples isolated from P3 mouse cochlea by dissociation and flow cytometry in an earlier study (Sinkkonen et al., 2011) (Figure 3A). We combined and normalized these P3 sample probe intensities [HC (*n* = 4) and SC (*n* = 2)] with those of our nine samples from E10.5 embryos described above (Supplemental File 1). We then performed principal component analysis to assess the transcriptome-wide similarities and differences between all of the samples (Figure 3B). Projection of the samples onto the first two principal components illustrates the inter-group relationship of otic sensory lineage populations and their segregation from non-otic samples.

To identify the most differentially expressed genes in the P3 hair cell and supporting cell populations, we compared individual probe intensity levels vs. the non-otic samples. The heat map of probe intensity values for the top 30 probes by OV rank across all samples shown in Figure 3C illustrates the diversity of gene expression profiles in these populations. As with the OV comparisons, HC fold Δ and SC fold Δ values were determined for each probe based on relative intensities between the HC or SC groups and the highest expressing non-otic group for a given probe (Table 2 and Supplemental File 1). In general, probe intensities and fold Δ values for the HC and SC groups showed a much wider range than the OV samples. This is expected due to the more differentiated state of neonatal HCs and SCs as compared to OV progenitors, as well as the more homogeneous makeup of the FACS purified P3 samples (Sinkkonen et al., 2011). Both HC and SC fold Δ values across all probes ranged from about 300 fold up regulated to about 300 fold down regulated (0.003 fold Δ). 684 probes had HC fold Δ values greater than 4, while 565 probes had SC fold Δ values greater than 4 (Supplemental File 1).

**Table 2:** **Comparison of P3 hair cell and supporting cell expression intensities to non-otic populations**.

**Gene**	**HC/Max non-otic**		**SC/Max non-otic**		**Otic consensus expression groups fold Δ > 4**	**Otic consensus score: sum of three otic ranks**	**Otic consensus rank:**
	**HC fold Δ**	**HC rank**	**SC fold Δ**	**SC rank**			
***Oc90***	**37.191532**	**36**	**133.68132**	**7**	**OV/HC/SC**	**44**	**3**
***Fbxo2***	**233.46231**	**3**	**209.10844**	**4**	**OV/HC/SC**	**9**	**1**
*Lmx1a*	1.071335	7460	2.1826358	1496	OV	8959	1220
*Krt1-23*	1.7182626	2426	7.448142	229	OV/SC	2659	259
*Spp1*	1.1694639	5352	1.0023074	16967	OV	22324	4802
*Cldn6*	1.8450933	2163	4.1609483	528	OV/SC	2697	264
Wfdc2^*^	3.2619166	904	14.833333	88	OV/SC	999	85
*Gata3*	17.05958	99	22.630722	39	OV/HC/SC	146	6
***Col9a2***	**64.73929**	**16**	**99.665436**	**13**	**OV/HC/SC**	**38**	**2**
*Arhgef19*	4.2322383	639	14.127765	93	OV/HC/SC	742	49
*Rbm35a*	2.1024952	1724	4.913607	432	OV/SC	2167	206
*Plekhb1*	28.310795	56	33.40806	25	OV/HC/SC	93	4
*Hs3st1*	4.9308343	517	22.05582	44	OV/HC/SC	574	33
*Rgcc*	4.3943467	600	9.030337	175	OV/HC/SC	789	56
*Tbx2*	4.6268463	553	3.1064396	849	OV/HC	1417	119
*Prss8*	5.4411964	442	12.075007	123	OV/HC/SC	581	35
*Sh3gl2*	10.561689	174	11.849528	125	OV/HC/SC	316	10
Wfdc2^*^	2.319336	1489	9.602433	161	OV/SC	1668	148
*Marveld3*	5.016969	502	9.084135	171	OV/HC/SC	692	45
*Vwa2*	1.0021158	10270	5.5677967	364	OV/SC	10654	1500
*Bdnf*	9.485823	208	1.0017089	19301	OV/HC	19530	3740
*Espn*	21.954702	79	1.1772411	5296	OV/HC	5397	653
*Plekha4*	4.1990533	647	6.789616	262	OV/HC/SC	932	75
*Fgf10*	13.839492	125	17.822388	64	OV/HC/SC	213	7
*Car4*	3.679346	25122	3.4888153	24946	OV	50093	18760
*Prr15*	6.245964	378	7.0138445	249	OV/HC/SC	653	42
*Col6a1*	1.2619317	21404	1.151628	5720	OV	27151	7232
*Six1*	5.8167586	403	5.891639	328	OV/HC/SC	759	52
*Socs2*	5.1087866	493	5.8204165	339	OV/HC/SC	861	64
*Myo7a*	28.857481	55	1.0124729	11440	OV/HC	11525	1651

The top 30 OV-ranked probes (as in Table 1) are shown here with HC and SC Fold Δs and rankings, otic consensus group designations, and otic consensus scores and ranks. Genes that appear more than once (^*^) are represented by multiple probe sets and those in bold are included in validation studies in later sections.

We next sought to identify genes with consensus of specific expression in the OV as well as SC and HC groups. Probes were evaluated for otic consensus expression based on fold change and rankings in the three otic categories. As an initial approach, a fold change threshold was used to assign otic consensus expression groups, based on a minimum change vs. max non-otic of 4-fold (Table 2 and Supplemental File 1). This analysis highlights the correlation of gene expression between the three otic populations and, in particular, shows that many genes strongly expressed in HCs and/or SCs are initially expressed quite early in otic development, showing elevated probe intensity in the OV. When we examined all probes ranked by OV fold Δ (Supplemental File 1) there is a notably high prevalence of probes with HC and/or SC consensus among the higher-ranked OV probes. Indeed, 17.4% of HC consensus probes and 25.5% of SC consensus probes (119/684 and 144/565 probes > 4-fold, respectively) are found within the top 500 probes by OV rank (Supplemental File 1). About half of the top 30-ranked OV probes shown in Table 2 are designated as having consensus expression above the threshold in all three otic populations (OV/HC/SC). Other probes are designated as consensus only for one or two otic groups (i.e., OV, OV/HC, or OV/SC). For example, *Lmx1a, Spp1, Car4*, and *Col6a1* are enriched greater than 4-fold in OV, but not in HCs or SCs, suggesting down regulation of these genes in both HC and SC branches of the otic sensory lineage during development. Probes with OV/HC consensus expression are also indicated in Table 2, and include *Tbx2, Bdnf, Espn*, and *Myo7a*. OV/SC consensus probes include *Krt1-23, Cldn6, Wfdc2*, and *Vwa2*. This analysis shows how assignment of expression groups based on fold change and *q*-value cutoffs can be useful in identify genes with expression in the different branches of the otic sensory lineage. However, outcomes from this type of analysis are highly modulated by fold change and statistical cutoffs (Dalman et al., 2012) so interpretations will vary and multiple methods of comparison are necessary.

To address this, we calculated otic consensus scores for each probe as the sum of the three otic ranks. All probe sets were ranked by otic consensus score values, which ranged from 9 to 77,083 (Supplemental File 1). Otic consensus scores and ranks illustrate the relative differences between total otic vs. non-otic expression of genes. Lower otic consensus scores represent more “otic lineage-specific” genes. A representation of all probes positioned by OV rank vs. otic consensus score illustrates their distribution across the dataset and highlights top otic consensus genes *Fbxo2, Col9a2*, and *Oc90* (Figure 3D). The broad range of otic consensus ranks among the 30 top OV genes shown in Table 2 is an indication of the diverse expression behavior of these genes in the later otic sensory lineages (HCs and SCs). The top 30 probes by OV consensus rank are listed in Table 3, with *Fbxo2, Col9a2*, and *Oc90* topping the list; all three with an OV rank less than 10 and OV consensus score below 50. Several of the probes in Table 3 represent well-known otic genes and some are listed more than once due to having multiple probe sets similarly ranked. This analysis also highlights genes that are likely expressed in the OV but were not particularly noted earlier due to the relatively low range of fold change values across all probes in OV vs. non-otic samples. For example, probes for *Btbd14a, Otolin, Kai1, Gjb2*, and *Ush1c*, have OV rankings from about 100–380 and OV fold changes from 2.5 to 1.5-fold, but all ranked well by otic consensus due to high fold changes in HC and SC groups combined with a reasonably high OV rank.

**Table 3:** **Top 30 probes by otic consensus rank**.

**Gene**	**OV/Max non-otic**		**HC/Max non-otic**		**SC/Max non-otic**		**Otic consensus**		
	**OV fold Δ**	**OV rank**	**HC fold Δ**	**HC rank**	**SC fold Δ**	**SC rank**	**Groups > 4-Fold Δ**	**Score: OV/SC/HC rank sum**	**Rank**
***Fbxo2***	**14.62200927**	**2**	**233.462206**	**3**	**209.1084184**	**4**	**OV/HC/SC**	**9**	**1**
***Col9a2***	**6.894714915**	**9**	**64.73931483**	**16**	**99.66541579**	**13**	**OV/HC/SC**	**38**	**2**
***Oc90***	**15.31678675**	**1**	**37.19151468**	**36**	**133.6813688**	**7**	**OV/HC/SC**	**44**	**3**
*Plekhb1*	5.946512058	12	28.31080827	56	33.40807691	25	OV/HC/SC	93	4
*S100a1*	2.908877684	83	118.1935475	8	106.9791597	11	HC/SC	102	5
*Gata3*	7.034011368	8	17.05957342	99	22.63071352	39	OV/HC/SC	146	6
Fgf10^*^	4.700220155	24	13.8394988	125	17.82238697	64	OV/HC/SC	213	7
Fgf10^*^	4.314080209	37	13.59648272	128	16.99160918	71	OV/HC/SC	236	8
*Btbd14a*	2.583935277	107	12.9935202	138	24.78606653	34	HC/SC	279	9
*Sh3gl2*	5.195890282	17	10.56169338	174	11.84953165	125	OV/HC/SC	316	10
Ap1m2^*^	4.401733497	34	16.98844137	100	8.629079783	190	OV/HC/SC	324	11
*Cldn3*	4.28578566	39	8.527481874	247	21.97050359	45	OV/HC/SC	331	12
*Otolin*	2.613673625	104	13.00452906	137	12.71711488	110	HC/SC	351	13
Faah^*^	3.024489321	75	9.804033123	197	12.65490027	111	HC/SC	383	14
Col2a1^*^	4.520159455	31	7.506419492	289	17.59092164	65	OV/HC/SC	385	15
*Cd9*	3.355323417	67	8.23098117	257	16.33853718	74	HC/SC	398	16
Col2a1^*^	4.232269996	40	7.827652714	271	14.83067647	89	OV/HC/SC	400	17
*Kai1*	1.755355331	295	19.26278326	88	20.30006957	55	HC/SC	438	18
*Matn1*	2.017228994	189	8.875465831	229	39.5214772	21	HC/SC	439	19
*Gal3st1*	2.881826605	86	10.55670683	175	8.735986943	186	HC/SC	447	20
Gjb2^*^	1.679839813	340	23.0501633	74	23.74169556	38	HC/SC	452	21
Ap1m2^*^	3.920424836	52	13.46897818	132	6.465748986	291	HC/SC	475	22
*Rab25*	3.077388814	73	8.457112871	251	9.816023707	156	HC/SC	480	23
*BC019731*	2.606254615	105	24.07525961	68	6.247095999	308	HC/SC	481	24
*Ush1c*	1.613973622	381	21.22888215	83	33.21939745	26	HC/SC	490	25
*Ltbp3*	1.657999303	354	14.0823195	122	35.97926226	23	HC/SC	499	26
*Cyb561*	2.949823565	80	10.6399017	172	6.880909012	255	HC/SC	507	27
Gjb2^*^	1.521293061	452	38.97023609	32	35.09867751	24	HC/SC	508	28
*Kcnk1*	4.298354088	38	6.723838937	332	10.01882675	151	HC/SC	521	29
Faah^*^	2.569395243	109	8.469575034	250	9.13829701	168	HC/SC	527	30

Genes that appear more than once (^*^) are represented by multiple probe sets and those in bold are included in validation studies in later sections.

### Analysis of *Fbxo2*/Fbx2 expression in the developing mouse

**Figure 4:**
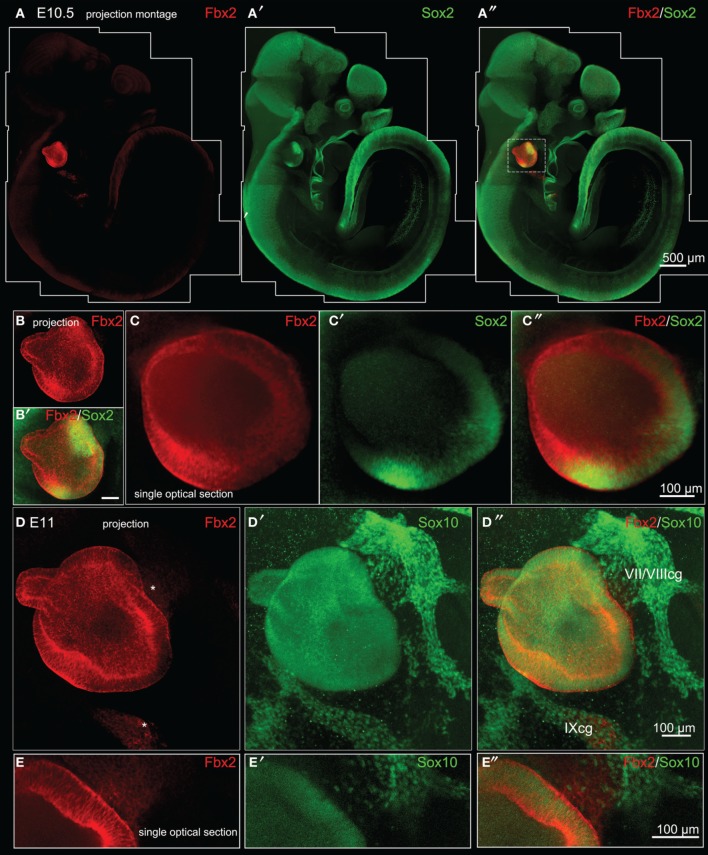
***Fbxo2*/Fbx2 expression in whole embryos at E10.5–E11**. Whole mount immunohistochemistry was performed with the antibodies indicated and embryos were imaged with confocal microscopy. Fbx2 expression is shown in red, with Sox2 at E10.5 **(A–C″)** and Sox10 at E11 **(D–E″)** in green. Fbx2 expression is strong in the otic vesicle epithelia and less intense expression is also noted (asterisks) in the neurons delaminating to the cochleovestibular and ninth cranial ganglia (VII/VIIIcg and IXcg).

**Figure 5:**
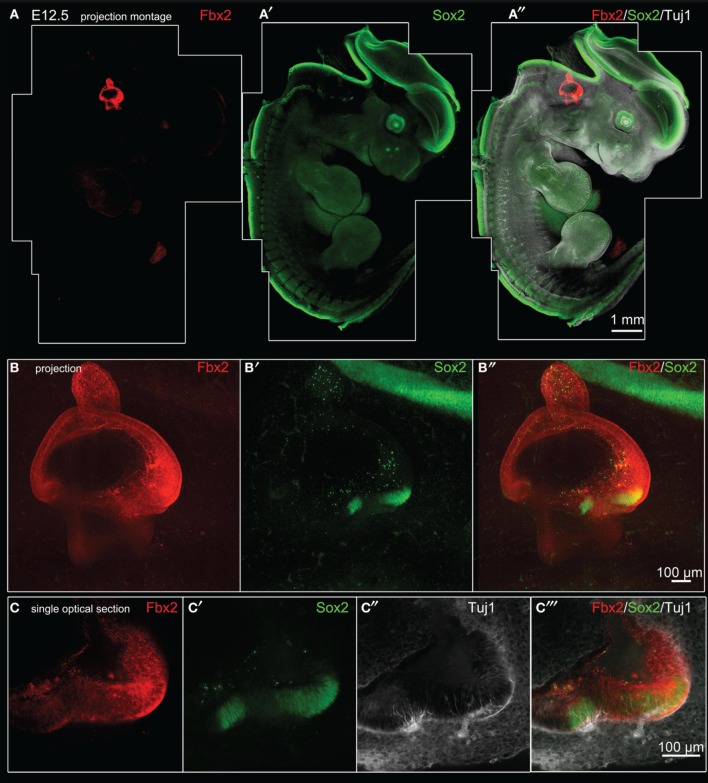
***Fbxo2*/Fbx2 expression in whole embryos at E12.5**. Whole mount immunohistochemistry was performed with the antibodies indicated and embryos were imaged with confocal microscopy. Fbx2 expression is shown in red, with Sox2 in green, and Tuj1 in white. **(A–A″)** Merged montages of maximum intensity z projections of confocal image stacks of a whole E12.5 embryo. **(B–B″)** show a projected stack of images through the otocyst region of the embryo shown in **(A)**. Sox2 expression is seen in two bright vestibular prosensory patches in the anterior region of the vestibule, while Sox2 expression in other prosensory domains (such as posterior vestibule or cochlea areas) are not visible in this projection due to lower intensity signal and attenuation from optical density of the tissue. **(C–C″)** Single optical sections through the anterior vestibular prosensory patches showing Fbx2-positive epithelia contain prosensory domains expressing bright Sox2 and containing Tuj1-labeled neurites.

**Figure 6:**
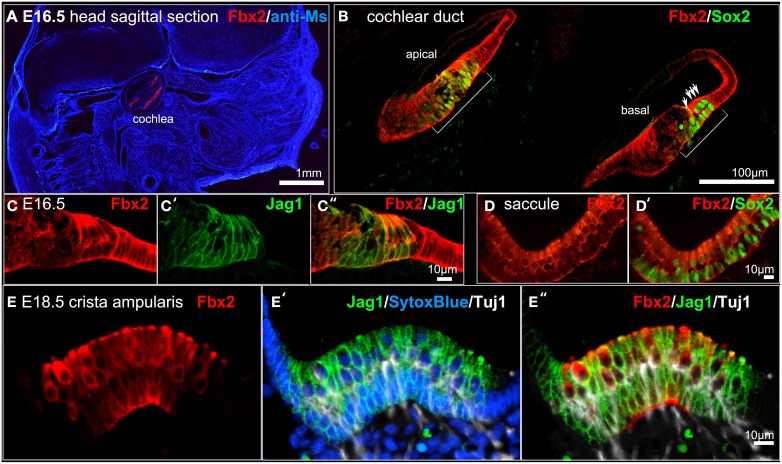
***Fbxo2*/Fbx2 expression in tissue sections at E16.5–E18.5**. Immunohistochemistry was performed with the antibodies indicated and embryos were imaged with confocal microscopy. Fbx2 expression is shown in red and additional antibodies or counter stains are shown in the colors indicated. **(A)** E16.5 sagittal head section immunolabeled for Fbx2 with anti-mouse IgG (blue) as a background stain. E16.5 cochlea **(B–C″)**, saccule **(D,D′)**, and E18.5 crista **(E–E″)** stained as indicated.

*Fbxo2* was the highest-ranking gene based on otic consensus score and was in the top five probes for each of the otic rankings (rank 2 in OVs, 3 in SCs, and 4 in HCs). *Fbxo2* encodes the F-box ubiquitin ligase F-Box 2 (Fbx2) and previous studies have shown that this protein is strongly expressed in juvenile and adult mouse and guinea pig cochlea (Thalmann et al., 1997; Henzl et al., 2001, 2004; Nelson et al., 2007). Mice with targeted deletion of *Fbxo2* exhibit age-related cochlear degeneration with hearing loss beginning at 2 months, and the only deficiency observed is the inner ear phenotype, suggesting specificity (Nelson et al., 2007). These studies indicate that Fbx2 functions to ensure protein quality control required for cochlear homeostasis. While expression of Fbx2 was described in the mature cochlea in the above studies, the developmental expression pattern has not been reported. Thus, we used an antibody against mouse Fbx2 to assess expression in the developing mouse, with whole mount preparations as well as tissue sections. We labeled whole embryos at early stages of otic development with anti-Fbx2 and anti-Sox2, optically cleared samples with the Scale method (Hama et al., 2011), and generated confocal z-stacks to assess expression *in toto* (Figures 4, 5). At E10.5, Fbx2 expression was robust throughout the OV including the Sox2+ prosensory and neurogenic domains and was undetectable in nearly all non-otic tissues (Figures 4A–C″). Sox2, which was not upregulated in the OV compared to the rest of the embryo based on the microarray, is broadly expressed in the developing central nervous system. An E11 otic vesicle stained for Fbx2 and Sox10 shows strong Fbx2 expression in the OV and lower levels of expression in delaminating cochleovestibular neuroblasts as well as the ninth cranial nerve ganglia (Figures 4D–E″). At E12.5, Fbx2 was detected only in the developing inner ear, where it was expressed throughout the membranous labyrinth (Figures 5A,B″), including prosensory epithelial regions that express Sox2 and contain Tuj1-labeled neurites (Figures 5C–C″). We stained for Fbx2 in tissue sections of embryos at E16.5 and E18.5 and co-labeled with antibodies to the prosensory/sensory domain markers Sox2 and Jag1 (Figure 6). Similar to earlier stages, expression of Fbx2 was strongly restricted to the otocyst-derived epithelium when compared broadly to the rest of the embryo in sagittal sections at E16.5 (Figure 6A). In the cochlea, Fbx2 was localized to the floor of the duct, including and flanking the sensory region marked by expression of Sox2 (Figure 6B) and Jag1 (Figures 6C–C″) and clearly expressed in both the young hair cells and supporting cells. In the vestibular epithelia including the saccule and crista, Fbx2 was also expressed in sensory regions, including hair cells and supporting cells (Figures 6D–E″).

### Analysis of *Col9a2*/Col9a2 expression in the developing mouse

**Figure 7:**
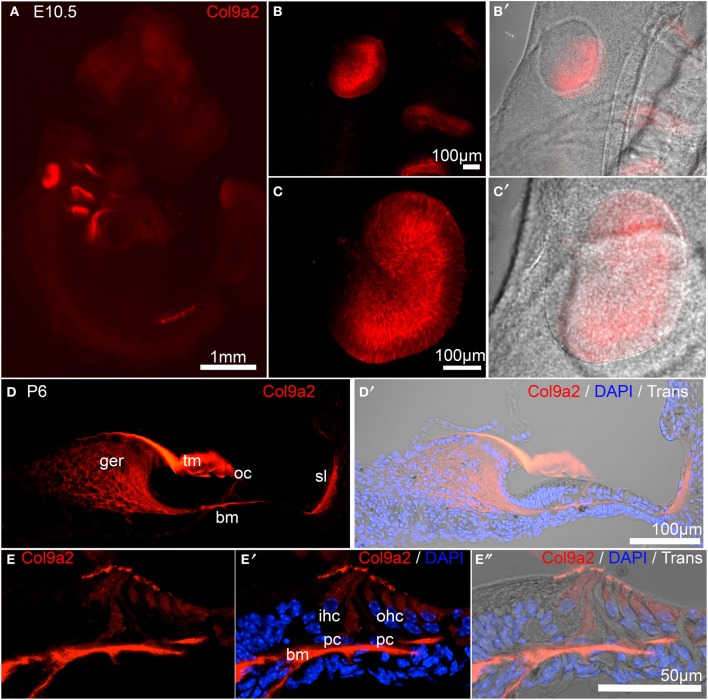
**Col9a2 expression in whole E10.5 embryo and neonatal cochlea**. Col9a2 immunoreactivity is shown in red in E10.5 whole embryo **(A)**. Higher magnification views show the pattern of reactivity in the OV and surrounding tissues **(B–C′)**. **(D,D′)** A cochlea cryosection at P6 shows Col9a2 immunoreactivity in the tectorial membrane, greater epithelial ridge/spiral limbus, organ of Corti, and spiral ligament. **(E–E″)** A higher magnification view of a similar section of the organ of Corti with the tectorial membrane removed (to better image the lower intensity signal in the organ of Corti) shows the Col9a2 signal in the basilar membrane, pillar cells and the developing tunnel of Corti, and inner and outer hair cells. Abbreviations: ger, greater epithelial ridge; tm, tectorial membrane; oc, organ of Corti; sl, spiral ligament; bm, basilar membrane.

*Col9a2* held the second otic consensus rank, with OV/HC/SC ranks of 9, 16, and 13, respectively. *Col9a2* encodes collagen alpha-2(IX), one of the three essential alpha chains of the collagen IX protein. A loss of function mutation in *Col9a2* was identified as a causative locus in autosomal recessive Stickler syndrome, characterized by hearing loss and ocular, skeletal, and orofacial abnormalities (Baker et al., 2011). Type IX collagen loss in mice (caused by absence of Col9a1, which leads to functional knockout of the entire collagen IX protein, Hagg et al., 1997), causes progressive hearing loss associated with abnormal integrity of collagen fibers in the tectorial membrane (Suzuki et al., 2005). The *Col9a1/2/3* genes were detected in adult mouse cochlea by RT-PCR and immunohistochemical analysis with a polyclonal antibody against type IX collagen indicated possible expression in the tectorial membrane (Asamura et al., 2005). Developmental expression of type IX collagen has not been investigated in the inner ear so we performed immunohistochemical analysis using a Col9a2-specific antibody to evaluate embryo-wide expression patterns (Figure 7). At E10.5, in a whole embryo preparation, Col9a2 immunofluorescence is prominent in the OV and is also visible in the branchial epithelia and pronephros (Figures 7A–B′). The signal is broad throughout most of the OV with reduced expression in the dorsal region (Figures 7C,C′). In P6 cochlea sections Col9a2 immunoreactivity is strong in the greater epithelial ridge (GER)/spiral limbus, tectorial membrane, organ of Corti, and spiral ligament (Figures 7D–E″). A closer look at the organ of Corti shows a strong signal in the basilar membrane as well as tunnel of Corti/pillar cell region and a less intense signal in hair cells (Figures 7E–E″).

### Analysis of *Oc90* expression in the developing mouse

**Figure 8:**
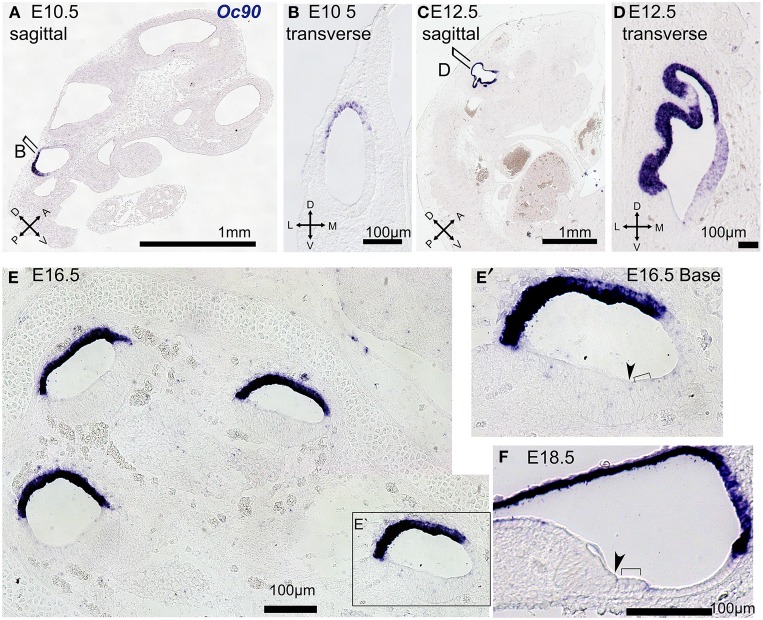
***Oc90* expression in tissue sections at E10.5–E18.5**. *In situ* hybridization was performed on paraffin sections at the indicated ages with antisense probe against *Oc90* (blue). E10.5 embryo specimens are shown in sagittal **(A)** and transverse views **(B)** and illustrate the dorsal and posterior expression domain of *Oc90* in the OV. At E12.5 the region of *Oc90* expression is expanded through most of the otic epithelium, save the most ventral region, as shown in sagittal **(C)** and transverse views **(D)**. **(E)** At E16.5 in a mid-modiolar section of the cochlea *Oc90* expression is strongest in the roof of the cochlear duct but is also present at lower signal levels in the thickened epithelium of the floor, as shown in higher magnification view in **(E′)**. **(F)** At E18.5 *Oc90* is strong in Reissner's membrane the stria vascularis and a lower level signal is also present in the organ of Corti and some non-sensory epithelial cells. In **(E′,F)**, inner hair cells are indicated with arrowheads and outer hair cells are indicated with brackets.

*Oc90* was the highest ranked probe by OV fold Δ and the third ranked by otic consensus score. *Oc90* (*Ocn-95, PLA2L*) encodes a 95-kDa secreted glycoprotein initially identified due to its abundant expression in the developing and adult inner ear and shown to be an essential organizer of the otoconial matrix (Wang et al., 1998; Verpy et al., 1999; Zhao et al., 2007, 2008). Consistent with earlier reports (Verpy et al., 1999), our *in situ* hybridization expression analysis indicated that Oc90 is highly specific to the otic vesicle at E10.5 (Figures 8A,B). At E12.5 *Oc90* expression was present throughout most of the otocyst with a strong signal in the dorsal regions and less intense signal noted in the ventral domains (Figures 8C,D). In the cochlear duct at E16.5 and E18.5 we observed a wide dynamic range of *Oc90* expression, with very intense signal in the roof of the cochlear duct (developing Reissner's membrane and stria vascularis) as well as weaker signal in the developing organ of Corti (Figures 8E,F). *In situ* hybridization is limited in capacity to evaluate expression signals across a wide dynamic range in regions of close proximity due to signal overdevelopment and saturation. Taken together with previous studies, our data suggest that expression of *Oc90* is high in the otic sensory lineage as compared to most genes. Within the inner ear, *Oc90* has a markedly higher expression in non-sensory otic cells, particularly in domains possibly derived from dorsal OV regions. The otic-specific expression and wide dynamic range of *Oc90* in the inner ear makes this an interesting gene for regulatory studies but its dominant expression is in otic non-sensory domains, making it less useful for otic sensory lineage studies.

## Discussion

We sought to systematically identify transcriptional features that distinguish the otic sensory lineage from other lineages of vertebrate development by employing a microarray approach to compare OV, HC, and SC populations with developmental non-otic tissues. Whole E10.5 embryos with the greater otic regions removed were used to represent a wide range of non-otic lineages from all three germ layers, and periotic tissues represented non-otic tissues proximally associated with the OV, such as dorsal neural tissues and periotic mesenchyme. The inclusion of two non-otic populations was effective for gauging dynamic range of probe intensity and identifying probes with expression domains outside of the inner ear. Multivariate analyses including PCA and Spearman's correlation demonstrated that samples from the three otic populations shared transcriptome-wide correlations in expression profiles that distinguish this lineage from non-otic populations. We further analyzed the microarray data to identify individual genes that are specifically expressed in the otic sensory lineage. Fold change comparisons of otic samples to the maximum expressing non-otic populations for each probe provided a conservative assessment of specificity and enabled ranking of probes across the dataset. Comparison of OV, HC, and SC fold changes for each probe allowed identification of otic consensus genes based on fold change thresholds. We developed otic consensus scores and rankings based on expression in individual otic categories to reduce the data to a single dimension to aid in identification of lineage-specific genes. The entire dataset is assembled in Supplemental File 1 as a searchable spreadsheet than can be sorted and further interrogated to suit the needs of individual readers.

A major motivation for this study was the previous lack of unambiguous otic sensory lineage markers, which has hindered advancement of regenerative and developmental studies. *In vitro* experiments aimed at producing otic progenitors and sensory epithelial cells from stem cells have had to rely on combinations of less specific markers to assay cell identities (Oshima et al., 2010; Koehler et al., 2013). Most current markers used to label potential early otic progenitor cells (i.e., Pax2/8, Gata3, Jag1, Sox2/9/10) are themselves transcription factors or signaling molecules involved in otic development. These highly conserved factors tend to be expressed in multiple embryonic lineages so coexpression of multiple markers has been used as an indication of otic identity. For example, Pax2 is expressed strongly in the otic vesicle at E10.5, but is also very abundant in the developing central nervous system and pronephros (Supplemental Figure 1A). On the other hand, Sox10 is expressed strongly in neural crest progenitors of the peripheral nervous system, but overlaps with Pax2 in the otic vesicle (Supplemental Figures 1A–A″). While coexpression of these two markers appears to be specific to the otic vesicle at this stage, this is a transient feature since Pax2 becomes downregulated in the otic sensory lineage soon after (Burton et al., 2004). Gata3 is one transcription factor that showed a high specificity to the otic sensory lineage based on our microarray analyses. This is consistent with the relatively restricted pattern of Gata3 expression observed in E10.5 mouse embryos (Supplemental Figure 1B). While the non-otic expression of Gata3 is not as prominent as with Pax2 or Sox10, domains of varying intensities are present in the central nervous system and pronephros, though generally weaker than in the otic vesicle. Coexpression of Gata3 and Fbx2 occurs exclusively in the otic vesicle at E10.5 (Supplemental Figures 1B–B″) and these markers both persist in the otic sensory lineage through later stages of development (Figure 6 and Luo et al., 2013).

Our study differs from most earlier microarray studies of the inner ear in its design as a comparative analysis of specific otic sensory lineage populations to a broad representation of non-otic lineages. Most of earlier analyses were conducted without comparative populations to other tissues. Many were designed to compare expression between wild type and mutant animals or between different tissues of the inner ear, such as at different ages or between the base and apex of the cochlea, or after insult (for review see Hertzano and Elkon, 2012). The most highly comprehensive microarray analysis of mouse inner ear morphogenesis included a total of 29 finely dissected inner ear samples in duplicate from E9 to E15 (Sajan et al., 2007). This study also included three samples designated as non-inner ear collected from areas in close proximity to the inner ear tissues pooled at E9, E9.5–10.5, and E11–15. These included neural tissue as well as mesenchyme, similar to the periotic tissue group used in this report. In another study aimed at identification of early otic-specific transcripts, cDNA subtractions of mouse otic vesicle against adult liver cDNA were used to identify candidate genes (Powles et al., 2004). Our use of whole embryos with the greater otic regions removed provided an inclusive representation of non-otic developmental lineages. While our findings indicate whole embryo-minus-otic tissue is an effective general non-otic reference population it has limitations in identifying genes expressed in smaller subsets of cells. We included periotic tissues as a separate non-otic population, which enabled us to gauge dynamic range of probes and evaluate the likelihood of non-otic expression domains based on differential expression between non-otic groups. Future studies may improve sensitivity of non-otic reference samples through further division of embryo tissue into a larger set of non-otic sample groups from smaller domains as well as from different stages of development.

Another feature distinguishing the current study is our use of data representing purified populations of HCs and SCs in combination with data from OV tissues to evaluate expression in distinct branches of the lineage before and after bifurcation of HC and SC fates. As the goal of this study was not to specifically search for genes that are exclusive for distinct otic lineages, we succeeded in the identification of markers that are much more restricted to the developing inner ear than those previously identified. The conditions that we chose for the top ranking genes were guided by restriction to the otic lineage at E10.5 and further by enrichment in hair cells and supporting cells in the neonatal cochlea.

Our analysis identified and ranked top otic sensory lineage-specific transcripts including *Fbxo2, Col9a2*, and *Oc90*. We verified otic-specific expression of these genes using immunohistochemistry and *in situ* hybridization, which revealed novel expression patterns and concurred with the array data. *Fbxo2*/Fbx2 showed the most striking pattern of specificity to the otic sensory lineage. Although Fbx2 expression has been reported in neuronal populations of the CNS (Eom et al., 2004) our analysis identified very few regions of expression outside of the inner ear and none with the robust intensity found in otic tissues. A likely explanation is that *Fbxo2* is expressed in some non-otic cell types, but at a very low level compared to the otic vesicle. The lack of any phenotype in *Fbxo2* null mice besides selective cochlear degeneration is evidence that this gene is functionally specific to the inner ear (Nelson et al., 2007). From our study it is clear that both at the transcript level and the protein level *Fbxo2* stands out from all other genes as a highly specific and robust marker of the otic sensory lineage. This feature elevates *Fbxo2* to an important gene for future studies of otic sensory lineage development and gene regulation. *Col9a2* also showed highly specific expression in the otic sensory lineage at both the transcript and protein levels. Compared to *Fbxo2* and *Oc90, Col9a2* is not as highly restricted to the otic sensory lineage as it is also expressed in branchial epithelia and pronephros (Figures 7A–B′). Col9a2 expression is initially robust in the OV epithelial cells and in the neonatal cochlea becomes organized largely in extracellular domains, including the tectorial and basilar membranes and the tunnel of Corti. We were unable to achieve adequate performance from a *Col9a2* probe for *in situ* hybridization in order to evaluate transcript levels in the organ of Corti, but this gene would be an interesting choice for future studies with a reporter mouse model. *Oc90* expression is highly specific to the inner ear and includes the sensory lineage populations but is actually expressed at even higher levels in non-sensory otic cells. The array data show *Oc90* at robust levels in the OV as well as HCs and SCs as compared to the non-otic samples. *In situ* hybridization also confirms highly specific expression of *Oc90* in the OV and, although expression is detectable in sensory progenitors of the organ of Corti, the strongest region of *Oc90* expression is found in non-sensory otic cells. This could be reflective of a very broad dynamic range of *Oc90* expression in the neonatal cochlea combined with a highly sensitive microarray probe, detecting the gene robustly in P3 HCs and SCs, and very low expression in the non-otic tissue groups resulting in a high rank for *Oc90* in HCs and SCs despite even higher expression of this gene in other cells. Beside *Fbxo2, Col9a2*, and *Oc90*, our analysis has revealed additional candidate novel otic sensory lineage genes and provided comparative expression data for quite a few well-known otic genes. Novel genes that show high-ranking consensus expression in the otic sensory lineage populations, such as *Plekhb1, Btbd14a, Ap1m2, Kai1*, and *Faah*, would be excellent candidates for further studies of gene expression and function in this lineage. Additionally the results of this study will aid in the design of experiments that require carefully selected lists of genes to assay for cell identity. For example, we used these data to inform the design of the 96-gene expression assay applied in recent work using single cell analysis of individual OV cells to reconstruct the otocyst and early neuroblast lineages *in silico* (Durruthy-Durruthy et al., 2014).

### Conflict of interest statement

The authors declare that the research was conducted in the absence of any commercial or financial relationships that could be construed as a potential conflict of interest.

## Supplementary material

Supplementary Figure 1 **E10.5 whole embryo immunohistochemistry**. **(A–A″)** Pax2 and Sox10 as indicated. **(B–B″)** Gata3 and Fbx2 as indicated.
