# Supplementary figures and images for: Identification and characterization of mouse otic sensory lineage genes
Source: Front Cell Neurosci. 2015 Mar 19;9:79. doi: 10.3389/fncel.2015.00079 (PMC4365716; doi:10.3389/fncel.2015.00079)

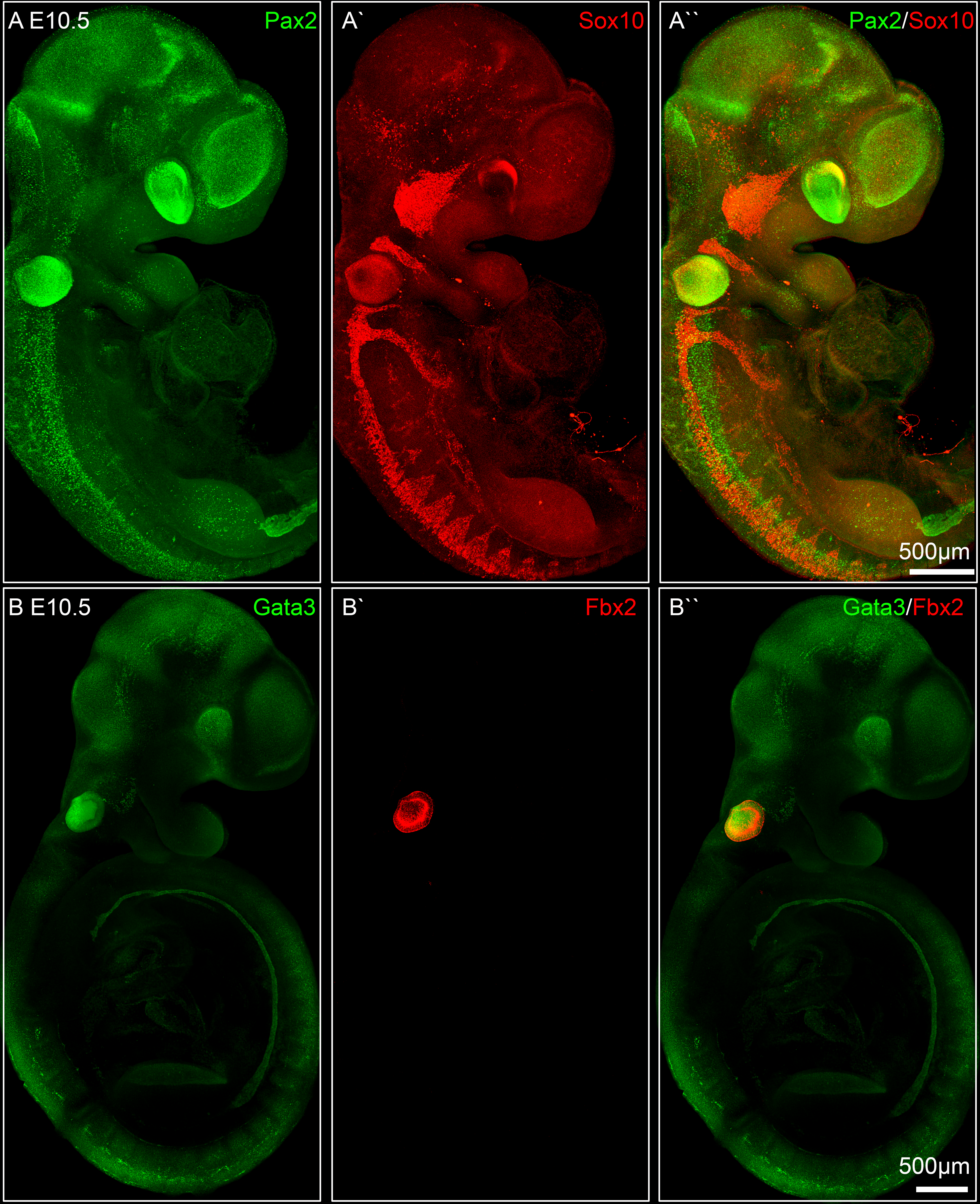

Supplement: Supplementary Figure 1 — E10.5 whole embryo immunohistochemistry. (A–A″) Pax2 and Sox10 as indicated. (B–B″) Gata3 and Fbx2 as indicated. [file Image1.TIF]
